# A Comparative pO_2_ Probe and [^18^F]-Fluoro-Azomycinarabino-Furanoside ([^18^F]FAZA) PET Study Reveals Anesthesia-Induced Impairment of Oxygenation and Perfusion in Tumor and Muscle

**DOI:** 10.1371/journal.pone.0124665

**Published:** 2015-04-22

**Authors:** Moritz Mahling, Kerstin Fuchs, Wolfgang M. Thaiss, Florian C. Maier, Martina Feger, Daniel Bukala, Maren Harant, Martin Eichner, Jörg Reutershan, Florian Lang, Gerald Reischl, Bernd J. Pichler, Manfred Kneilling

**Affiliations:** 1 Werner Siemens Imaging Center, Department of Preclinical Imaging and Radiopharmacy, Eberhard Karls University Tübingen, Röntgenweg 13, 72076 Tübingen, Germany; 2 Department of Dermatology, Eberhard Karls University Tübingen, Liebermeisterstraße 25, 72076 Tübingen, Germany; 3 Department of Radiology, Eberhard Karls University Tübingen, Hoppe-Seyler-Straße 3, 72076 Tübingen, Germany; 4 Department of Physiology, Eberhard Karls University Tübingen, Gmelinstrasse 5, 72076 Tübingen, Germany; 5 Institute for Clinical Epidemiology and Applied Biometry, Eberhard Karls University Tübingen, Silcherstraße 5, 72076 Tübingen, Germany; 6 Department of Anesthesiology and Intensive Care Medicine, Eberhard Karls University Tübingen, Hoppe-Seyler-Straße 3, 72076 Tübingen, Germany; Wayne State University, UNITED STATES

## Abstract

Tumor hypoxia can be identified by [^18^F]FAZA positron emission tomography, or invasively using oxygen probes. The impact of anesthetics on tumor hypoxia remains controversial. The aim of this comprehensive study was to investigate the impact of isoflurane and ketamine/xylazine anesthesia on [^18^F]FAZA uptake and partial oxygen pressure (pO_2_) in carcinoma and muscle tissue of air- and oxygen-breathing mice.

**Methods:**

CT26 colon carcinoma-bearing mice were anesthetized with isoflurane (IF) or ketamine/xylazine (KX) while breathing air or oxygen (O_2_). We performed 10 min static PET scans 1 h, 2 h and 3 h after [^18^F]FAZA injection and calculated the [^18^F]FAZA-uptake and tumor-to-muscle ratios (T/M). In another experimental group, we placed a pO_2_ probe in the tumor as well as in the *gastrocnemius* muscle to measure the pO_2_ and perfusion.

**Results:**

Ketamine/xylazine-anesthetized mice yielded up to 3.5-fold higher T/M-ratios compared to their isoflurane-anesthetized littermates 1 h, 2 h and 3 h after [^18^F]FAZA injection regardless of whether the mice breathed air or oxygen (3 h, KX-air: 7.1 vs. IF-air: 1.8, p = 0.0001, KX-O_2_: 4.4 vs. IF-O_2_: 1.4, p < 0.0001). The enhanced T/M-ratios in ketamine/xylazine-anesthetized mice were mainly caused by an increased [^18^F]FAZA uptake in the carcinomas. Invasive pO_2_ probe measurements yielded enhanced intra-tumoral pO_2_ values in air- and oxygen-breathing ketamine/xylazine-anesthetized mice compared to isoflurane-anesthetized mice (KX-air: 1.01 mmHg, IF-air: 0.45 mmHg; KX-O_2_ 9.73 mmHg, IF-O_2_: 6.25 mmHg). Muscle oxygenation was significantly higher in air-breathing isoflurane-anesthetized (56.9 mmHg) than in ketamine/xylazine-anesthetized mice (33.8 mmHg, p = 0.0003).

**Conclusion:**

[^18^F]FAZA tumor uptake was highest in ketamine/xylazine-anesthetized mice regardless of whether the mice breathed air or oxygen. The generally lower [^18^F]FAZA whole-body uptake in isoflurane-anesthetized mice could be due to the higher muscle pO_2_-values in these mice compared to ketamine/xylazine-anesthetized mice. When performing preclinical in vivo hypoxia PET studies, oxygen should be avoided, and ketamine/xylazine-anesthesia might alleviate the identification of tumor hypoxia areals.

## Introduction

Tumor hypoxia is one of the major hallmarks of malignant tumors associated with tumor progression [1, 2]. Hypoxic tumors are more resistant to radiotherapy, and patients with hypoxic tumors have a poorer prognosis [1]. In the clinical context, tumor hypoxia assessment employing the widely used positron emission tomography (PET) tracer ^18^F-fluoro-misonidazole ([^18^F]FMISO) is helpful for physicians in planning and adjusting radiotherapy [3]. A commonly used method to validate such imaging-based, non-invasive hypoxia measurements in the preclinical setting is an oxygen probe system to obtain invasive *in vivo* partial oxygen pressure (pO_2_) measurements [4]. A general and important requirement for these *in vivo* studies in rodents is unconsciousness of the animals achieved by anesthesia. However, anesthetics can significantly alter the physiology [5], change the pharmacokinetics of the PET tracer and can thereby bias tumor hypoxia measurements.

Isoflurane (IF) inhalation anesthesia or a combination of ketamine and xylazine (KX) are frequently used in preclinical studies [6]. The inhalation agent IF is a gamma-aminobutyric acid A (GABA_A_) receptor agonist that can be easily administered and induces safe and fast anesthesia with sedation and relaxation but negligible analgesia [7]. In contrast, ketamine is an *N*-methyl-D-aspartate (NMDA) receptor antagonist that induces a “dissociative anesthesia” after intraperitoneal injection [8]. Ketamine is usually co-administered with xylazine, an α_2_-adrenoreceptor agonist that contributes strong sedation and muscle relaxation [9]. Moreover, by co-administering with ketamine, the side effects of xylazine, such as reduced cardiac output, can be limited [10]. Despite their well-known effects on mammalian physiology, anesthetics can impair metabolic parameters (**S1 Table**). Xylazine both reduces cardiac output and vascular tonus and induces hyperglycemia [11–13]. All of these agents have specific effects on the cardiovascular system, as recently summarized by our group and others [7, 14]. Thus, for PET studies, it is important to know the anesthesia-specific side effects on basic physiological parameters such as heart rate, breathing rate and blood pressure. A detailed review of the effects of the individual anesthetic agents and it’s combinations on murine physiology is available as **S1 Table.**

Among hypoxia PET tracers, [^18^F]FAZA is a 2-nitroimidazole compound with faster diffusion into the tissue and an increased tumor-to-muscle ratio (T/M) compared to the commonly used tracer [^18^F]FMISO [15–17]. Another sophisticated and accurate method of detecting the oxygenation of tumor or muscle tissue is an invasive fluorescence measurement that assesses the tissue pO_2_ [4]. In contrast to non-invasive PET imaging, invasive optical fluorescence-based pO_2_ systems have a higher temporal resolution and provide absolute pO_2_-values. Our optical fluorescence-based system even enabled simultaneous measurements of the partial oxygen pressure and perfusion within the carcinoma or muscle tissue. However, compared to the non-invasive nature of PET imaging, pO_2_ sensors are highly invasive, have spatial constraints and allow only for measurements of superficial tumors; furthermore, their applicability for the measurement of tumor hypoxia in patients is limited.

Previous studies investigating the impact of anesthetics on hypoxia imaging have produced controversial results [15, 18, 19]. Thus, the impact of the commonly used anesthetics IF and KX as well as the use of different breathing gases on tumor and muscle oxygenation remains controversial. Additionally, to date, no study comparing the impact of the anesthetics that are mostly used for non-invasive imaging is available to provide a comprehensive overview of the general physiological condition of the awake and anesthetized animal, the differential impact of the anesthesia on the imaging-assessed and on the invasive probe-assessed hypoxia quantification. However, the use of different anesthetic agents and breathing gases might impair the reliability and comparability of *in vivo* PET imaging studies, and agents should therefore be selected carefully. This knowledge is especially crucial when comparing results among different research facilities. Thus, the aim of this study was to analyze and compare the different effects of IF and KX anesthesia as well as air- or oxygen-breathing on *in vivo* [^18^F]FAZA uptake in CT26 tumor and muscle tissue. Additionally, we performed invasive tissue oxygen tension (pO_2_) and perfusion measurements in CT26 tumors and muscle tissue to cross-validate our [^18^F]FAZA-PET results and investigated the different effects of IF and KX anesthesia on heart and breathing rates and blood pressure.

## Material and Methods

### 2.1 CT26 colon carcinoma mouse model

Female BALB/c mice (Charles River Laboratories International Inc., Wilmington, Massachusetts, USA) aged 8–13 weeks were investigated according to the German animal protection law. The animal use and care protocols were approved by the responsible Committee on the Ethics of Animal Experiments of the *Regierungspräsidium Tübingen*. CT26 mouse colon carcinoma cells were provided by Prof. Dr. med. Ralph Mocicat (Institute of Molecular Immunology, German Research Center for Environmental Health, Munich, Germany) and were cultured as previously described [20, 21]. We subcutaneously injected 0.5 x 10^6^ CT26 colon carcinoma cells in 200 μL NaCl into the left upper flank to induce a palpable tumor [21, 22]. Investigations were performed at day 10–14 after inoculation. The overall mean tumor volume was 298.3 mm^3^ (standard deviation (±) 45.0 mm^3^). The mean tumor volumes in the experimental groups were not significantly different (mean ± SD: IF-air: 279 ± 51.5 mm^3^, IF-O_2_: 283 ± 45.7 mm^3^, KX-air: 308 ± 43.8 mm^3^, KX-O_2_: 321 ± 28.5 mm^3^).

### 2.2 Anesthesia and breathing gas mixtures

Inhalation anesthesia with IF (Forene, Abbott Labs, Baar, Switzerland) was applied using a calibrated vaporizer (Vetland, Louisville, KY, USA) to induce and maintain anesthesia with 3 vol.% and 1.5 vol.% IF, respectively. KX anesthesia was induced by an intraperitoneal injection of 100 mg/kg ketamine (Ratiopharm, Ulm, Germany) and 5 mg/kg xylazine (Rompun, Bayer HealthCare, Leverkusen, Germany) in a volume of 0.1 mL / 10 g body weight. To maintain steady anesthesia, 25% of the initial dose was administered every 20 min.

We applied two different breathing gas mixtures (GSM-3 Gas Mixer, CWE Inc., Ardmore, PA, USA) using a closed box or a specially prepared nose cone at a constant flow of 1.5 L / min. The gas mixer provided 21% O_2_ / 79% nitrogen (N_2_; air) or 100% O_2._ For all measurements, we compared mice under IF or KX anesthesia breathing air or oxygen, i.e., we studied four experimental groups: IF-air, IF-O_2_, KX-air and KX-O_2_. The incubation with the specific breathing gas started 30 min prior to the induction of anesthesia or to the start of the measurement and was maintained throughout the whole investigation and during the PET scans.

### 2.3 Non-invasive [^18^F]FAZA PET imaging

[^18^F]FAZA was synthesized at the Department of Preclinical Imaging and Radiopharmacy as described previously [23]. Twenty minutes after the induction of anesthesia, mice were intravenously injected with 13.0 (± 0.09) MBq [^18^F]FAZA through the tail vein as described [20] (**Table 1**). At 55 min, 115 min and 175 min post-injection (p.i.), we placed two mice head-to-head in a calibrated Inveon microPET scanner (Siemens Medical Solutions, Knoxville, TN, USA) with a spatial resolution of 1.4 mm at the center field of view and an axial field of view (FoV) of 12.7 cm [24]. We used Inveon Acquisition Workplace (Siemens) software for PET data acquisition. Throughout the whole investigation, mice were held at a constant temperature of 37° C. Static 10-minute PET scans were performed 1 h, 2 h and 3 h p.i and reconstructed with the iterative two-dimensional ordered subset expectation maximization (OSEM2D) algorithm with 4 iterations and 16 subsets. Additional, 3 h dynamic scans were performed with air-breathing mice that were anesthetized with IF or KX, accordingly (n = 4). Both groups were injected with a comparable activity of [^18^F]FAZA (IF-air: 12.37 ± 0.12 MBq, KX-air: 12.21 ± 0.12 MBq). We analyzed the images with PMOD software (PMOD Technologies Ltd., Adliswil, Switzerland). Spheres with a radius of 2.0 mm were drawn around the tumor voxel with the maximum uptake as well as in the contralateral muscle tissue. All values were corrected for the injected dose and the decay of ^18^F. We calculated the relative [^18^F]FAZA injected dose per gram (%ID/g), the tracer clearance (%ID/g_3h_ / %ID/g_1h_) and the tumor-to-muscle ratios.

**Table 1:** Investigation protocol for [^18^F]FAZA PET measurements and T/M-ratios.

Time →	-50 min	-20 min	0 min	1 h [median (IQR)]	2 h [median (IQR)]	3 h [median (IQR)]
**Action →**	*Breathing gas start and maintenance*	*Anesthesia induction*	*Tracer injection*	*10 min PET-scan*	*10 min PET-scan*	*10 min PET-scan*
	Air	isoflurane	[^18^F]FAZA	1.2 (1.1–1.4)	1.3 (1.0–1.9)	1.8 (1.1–2.9)
	Air	ketamine/xylazine	[^18^F]FAZA	2.3 (1.7–2.5)	4.2 (2.3–6.5)	7.1 (3.6–10.0)
	Oxygen	isoflurane	[^18^F]FAZA	1.2 (1.1–1.3)	1.4 (1.2–1.6)	1.4 (1.1–2.3)
	Oxygen	ketamine/xylazine	[^18^F]FAZA	1.7 (1.4–2.0)	2.7 (2.2–3.5)	4.4 (3.0–5.3)

### 2.4 Invasive pO_2_-probe measurement

Ten minutes after the induction of anesthesia, we placed a calibrated pO_2_ / perfusion probe (BF/OFT/E-probe, Oxford Optronix Ltd., Oxford, U.K.) in the center of the tumor and in the contralateral *gastrocnemius* muscle (depth 4 mm). The probe was retracted by 1 mm after each anterograde movement to minimize tissue compression. Data acquisition started 10 min after the last probe manipulation and was conducted for 60 min (= baseline measurement). Mice in all of the groups (**Table 1**) were then challenged with 10 min of oxygen-breathing followed by 10 min of air-breathing. Data were analyzed with the OxyLite system (Oxford Optronix Ltd.).

### 2.5 Comprehensive monitoring of the animal physiology

The heart rate was measured with the BioVet CT 1 System (m2m Imaging Corp, Cleveland, OH, USA). Three electrocardiography electrodes were attached to the paws and to one hind leg. The breathing rate of the anesthetized mice was recorded using a breathing pad (m2m Imaging Corp) placed underneath the animals.

Additionally, the breathing rate of conscious animals was measured by a whole-body plethysmograph (PLY 311, EMMS, Bordon, U.K., **S1 Fig**) connected to a spirometer (ML 141 Spirometer, AD Instruments GmbH, Spechbach, Germany) and a gas mixer (GSM-3 Gas Mixer). After a habituation period of 15 min, we recorded the breathing signal for 20 min while the mice were monitored by a camera. The data were analyzed with an in-house programmed *MATLAB* routine (version R2013a, MathWorks Inc., Natick, MA, USA). Sniffing, moving and sleeping periods were excluded from the analysis.

Non-invasive systolic blood pressure measurements were obtained using the *tail-cuff* method (IITC life sciences, Los Angeles, CA, USA). Ten minutes after the induction of anesthesia, the blood pressure was measured five times during a period of 5 min to ensure the reproducibility and the robustness of the method.

### 2.6 Data recording and statistical analysis

All data except for the PET data were recorded with PowerLab (PowerLab 16/35, AD Instruments GmbH) and viewed with LabChart (LabChart 7.2, AD Instruments GmbH). Statistical analysis was performed using JMP software (Version 11.1.1, 64 bit, 2012 SYS Institute Inc., Cary, NC, USA). All graphs were designed with GraphPad Prism 6 for Windows (Version 6.01, GraphPad Software, Inc., La Jolla, CA, USA). The results are indicated as the median and interquartile ranges (IQR) and are graphically shown as Tukey box-and-whisker plots. For statistical testing, the data were natural log-transformed and compared using a t-test assuming equal variances. The global α-level was set to 0.05. To correct for multiple comparisons, we adjusted the significance level locally, i.e., for each data set, according to the *Bonferroni-Holm* method [25]. Statistically significant differences are indicated by “*”, and p-values below the unadjusted α-level of 0.05 but failing the local significance level determined by the *Bonferroni-Holm* method are indicated by “n.s.”.

## Results

### 3.1 Isoflurane and ketamine/xylazine anesthesia differentially impact [^18^F]FAZA PET imaging

**Fig 1:**
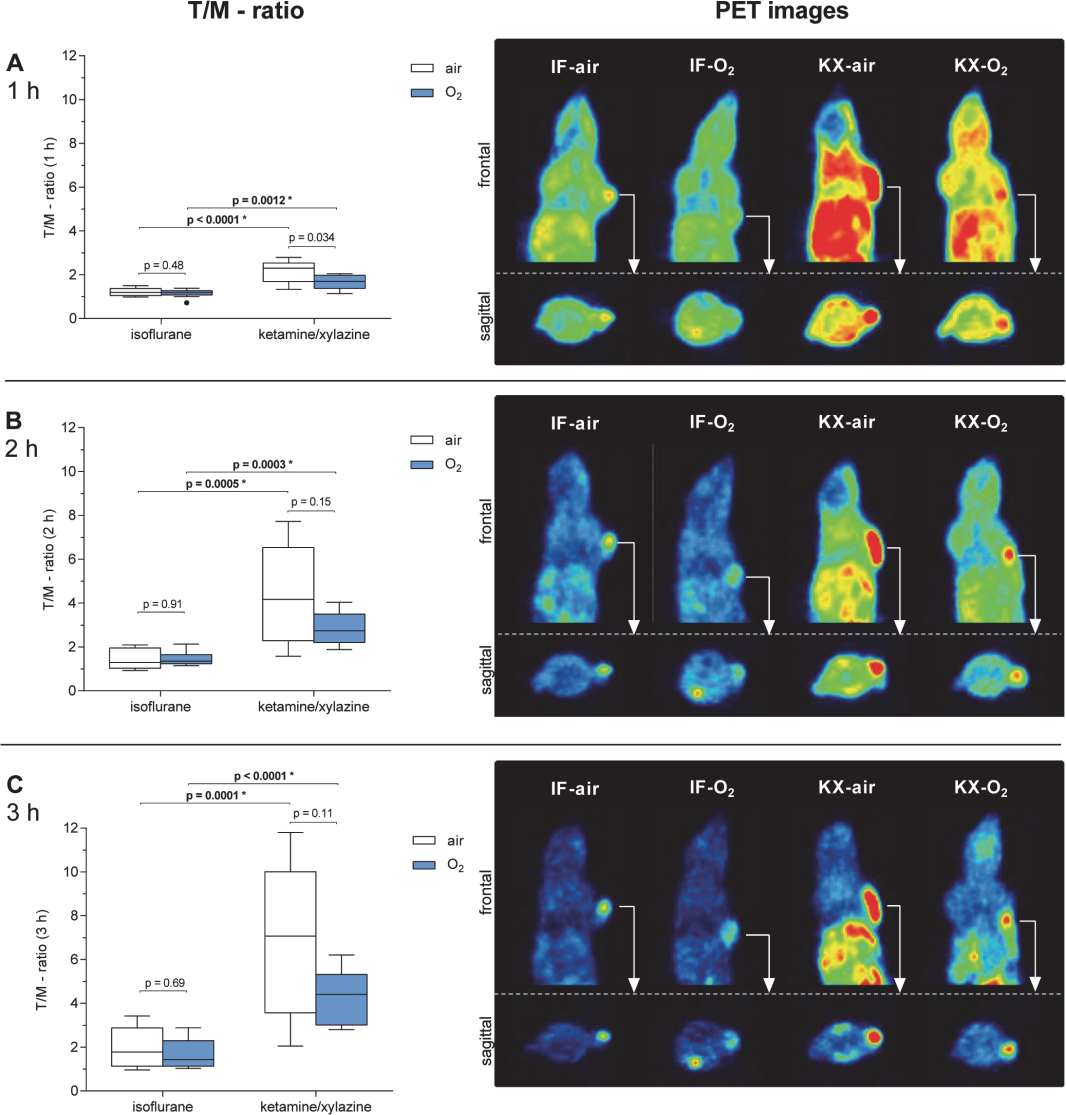
T/M-ratios (n = 7–12, left) and representative PET images at 3 h p.i. of mice anesthetized with isoflurane (IF) or ketamine/xylazine (KX) while breathing air or O_2_. Images and graphs show the static PET evaluation at 1 h (first row), 2 h (second row) and 3 h (third row) after [^18^F]FAZA injection. The image plane is positioned to display the “hottest” tumor voxel. p-values without asterisks (*) were not significant (after correcting for multiple testing).

To determine whether IF- or KX-anesthesia impact [^18^F]FAZA PET imaging, we calculated the [^18^F]FAZA PET T/M-ratios of spontaneously air- or oxygen-breathing, IF- or KX-anesthetized CT26 mice 1 h, 2 h and 3 h after [^18^F]FAZA injection (**Table 1 and Table 2A**). We observed significant, up to 3.5-fold, lower T/M-ratios in IF-anesthetized mice compared to KX-anesthetized mice 1 h (IF-air: 1.2 vs. KX-air: 2.3, p < 0.0001*, IF-O_2_: 1.2 vs. KX-O_2_: 1.7, p = 0.0012*, **Fig 1A**), 2 h (IF-air: 1.3 vs. KX-air: 4.2, p = 0.0005*, IF-O_2_: 1.4 vs. KX-O_2_: 2.7, p = 0.0003*, **Fig 1B**) and 3 h (IF-air: 1.8 vs. KX-air: 7.1, p = 0.0001*, IF-O_2_: 1.4 vs. KX-O_2_: 4.4, p < 0.0001*, **Fig 1C**) after [^18^F]FAZA injection, indicating increased tumor hypoxia due to combined KX-anesthesia (n = 7–12). Additionally, mice under IF-anesthesia displayed a lower whole-body uptake 1 h, 2 h and 3 h after [^18^F]FAZA injection compared to KX-anesthetized animals regardless of whether the mice breathed air or oxygen (PET images in **Fig 1A, 1B and 1C**).

**Table 2:** PET, invasive measurements and physiology values (median and IQR).

	A) PET			B) Invasive measurements				C) Physiology		
Group	T/M 1 h	T/M 2 h	T/M 3 h	Tumor pO_2_ [mmHg]	Muscle pO_2_ [mmHg]	Tumor perfusion [BPU]	Muscle perfusion [BPU]	Heart rate [/min]	Breathing rate [/min]	Blood pressure [mmHg]
Isoflurane—air	1.2 (1.1–1.4)	1.3 (1.0–1.9)	1.8 (1.1–2.9)	0.45 (0.35–0.47)	56.85 (49.08–59.23)	503 (272–559)	345 (294–628)	376 (352–394)	133 (127–147)	86 (85–92)
isoflurane—oxygen	1.2 (1.1–1.3)	1.4 (1.2–1.6)	1.4 (1.1–2.3)	6.25 (0.39–43.57)	95.64 (90.02–127.86)	718 (397–1173)	1122 (546–2279)	397 (391–421)	149 (137–151)	84 (81–88)
ketamine/xylazine—air	2.3 (1.7–2.5)	4.2 (2.3–6.5)	7.1 (3.6–10.0)	1.01 (0.67–1.26)	33.79 (26.86–42.53)	340 (266–496)	514 (365–619)	225 (214–263)	213 (190–224)	80 (72–85)
ketamine/xylazine—oxygen	1.7 (1.4–2.0)	2.7 (2.2–3.5)	4.4 (3.0–5.3)	9.73 (2.49–20.01)	83.48 (72.22–105.79)	636 (301–1055)	582 (386–662)	199 (173–216)	174 (167–193)	83 (77–86)

**Fig 2:**
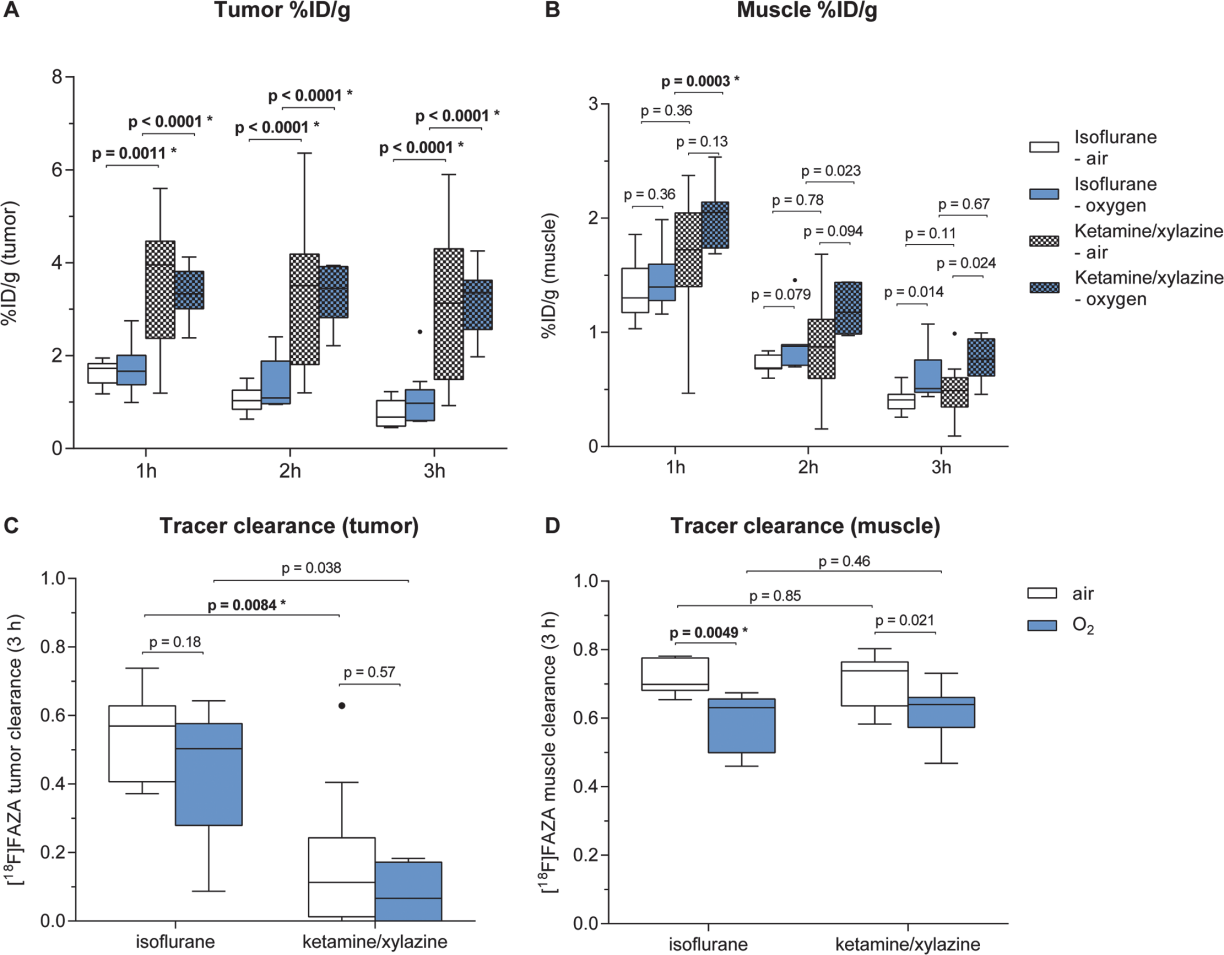
[^18^F]FAZA %ID/g in the tumor (A) and the muscle (B) at 1 h, 2 h and 3 h after [^18^F]FAZA injection (Tukey box and whisker plot, outliers represented as •). [^18^F]FAZA clearance from the tumor (**C**) and muscle (**D**) comparing 3 h to 1 h after tracer injection. N = 7–12. p-values without asterisks (*) were not significant (after correcting for multiple testing).

When observing different T/M-ratios in experimental groups, it is important to determine whether the differences are caused by an altered %ID/g uptake in the tumor or by an altered %ID/g uptake in the muscle. The decreased T/M-ratios in IF-anesthetized mice compared to their KX-anesthetized littermates were mainly caused by decreased [^18^F]FAZA %ID/g uptake values in the CT26 carcinomas (**Fig 2A,** values in **S2 Table**), whereas differences in the %ID/g values in the muscle tissue between IF- and KX-anesthetized mice were smaller (**Fig 2B**). Additionally, the [^18^F]FAZA clearance from the carcinomas between 1 h and 3 h after [^18^F]FAZA injection was higher in animals anesthetized with IF compared to their KX-anesthetized littermates (IF-air: 0.57 (0.41–0.63) vs. KX-air: 0.11 (0.01–0.24), p = 0.0084*, IF-O_2_: 0.50 (0.28–0.58) vs. KX-O_2_: 0.07 (-0.06–0.17), p = 0.038 n.s., n = 7–12, **Fig 2C**).

### 3.2 Air- and oxygen-breathing differentially impact [^18^F]FAZA uptake in muscle tissue

**Fig 3:**
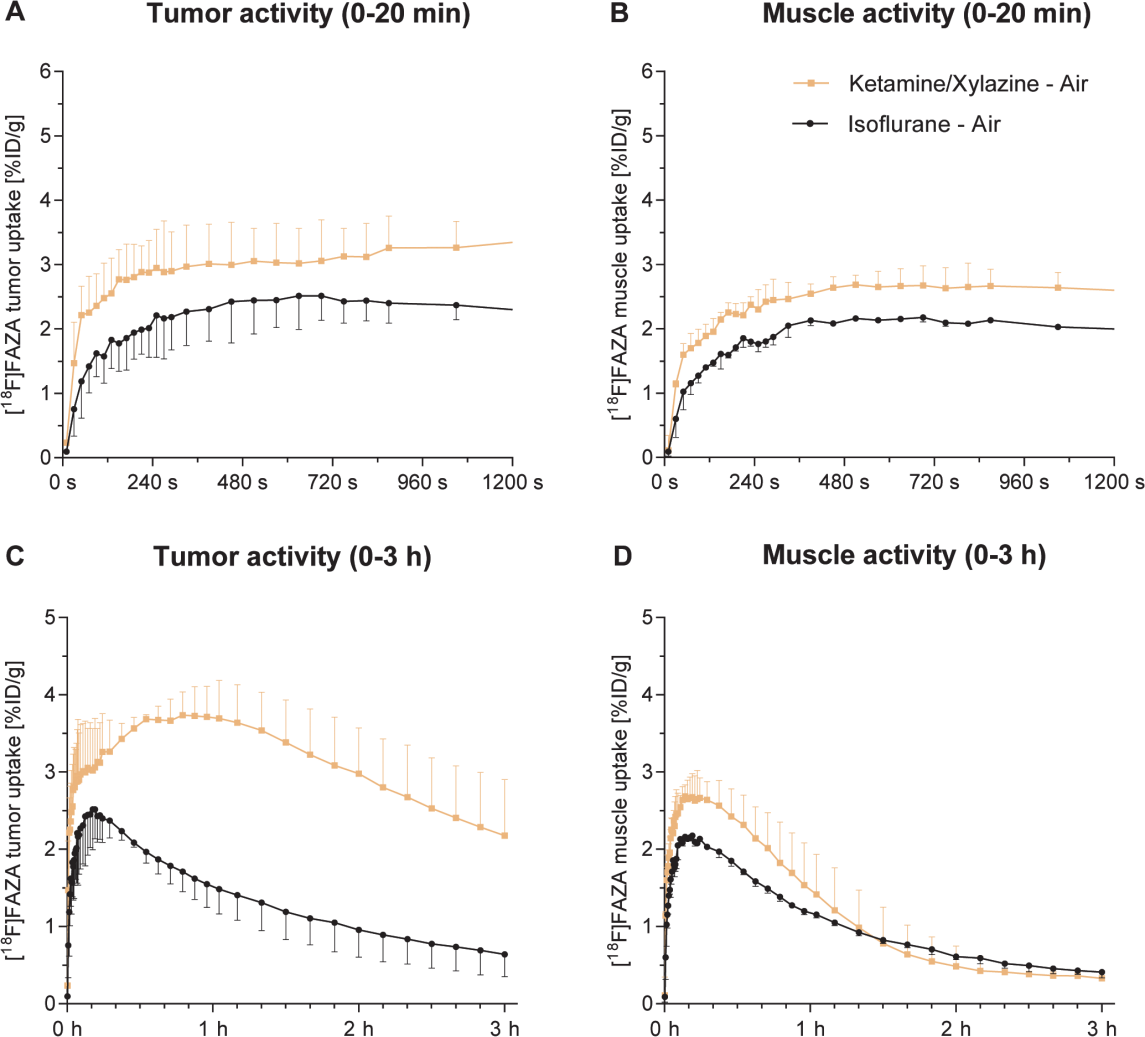
Dynamic uptake of [^18^F]FAZA %ID/g in tumor (A, C) and muscle (B, D) (median and IQR). **A** and **B** show the initial perfusion phase (0–20 min), and **C** and **D** show the uptake during 0–3 h (n = 4).

As expected, we observed lower T/M-ratios in oxygen-breathing KX-anesthetized mice compared to air-breathing mice at 1 h (KX-O_2_: 1.7 vs. KX-air: 2.3, **Fig 1A**), 2 h (KX-O_2_: 2.7 vs. KX-air: 4.2, **Fig 1B**) and 3 h (KX-O_2_: 4.4 vs. KX-air: 7.1, **Fig 1C**), although the differences were not statistically significant. The reduced T/M-ratios in the oxygen-breathing mice may have resulted from an anesthesia-independent increase in the muscle uptake of [^18^F]FAZA in oxygen-breathing IF- or KX-anesthetized mice compared to air-breathing mice, especially 2 h and 3 h after [^18^F]FAZA injection (**Fig 2B**) and not by a decreased uptake of [^18^F]FAZA by the tumors (**Fig 2A**). These observations are further supported by the reduced tracer clearance from the muscle of oxygen-breathing compared to air-breathing mice regardless of whether the mice underwent IF or KX anesthesia (**Fig 2D**). Interestingly, we did not observe differences in the T/M-ratios between IF-anesthetized oxygen- or air-breathing mice (1 h: IF-O_2:_ 1.2 vs. IF-air: 1.2, 2 h: IF-O_2:_ 1.4 vs. IF-air: 1.3, 3 h: IF-O_2:_ 1.4 vs. IF-air: 1.8, **Fig 1A–1C**).

To uncover differences in the kinetics of [^18^F]FAZA uptake and washout, we performed additional, dynamic [^18^F]FAZA-PET studies (**Fig 3**, n = 4) in air breathing KX- and IF-anesthetized mice. [^18^F]FAZA time activity curves (TACs) revealed no perfusion peak immediately after tracer injection in KX-anesthetized and in IF-anesthetized mice. However, we found higher [^18^F]FAZA uptake in the CT26 colon carcinomas and muscle tissue of KX-anesthetized mice than in those of their IF-anesthetized littermates during the first 20 minutes after [^18^F]FAZA injection (**Fig 3A and 3B**). Accordingly, 3 h TACs revealed higher [^18^F]FAZA uptake in tumors of KX-anesthetized mice than in those of IF-anesthetized mice (**Fig 3C**). The area under the curve (AUC) of the %ID/g in the tumor was significantly higher in KX-anesthetized animals (14,422 (11,884–17,329) %ID/g) than in their IF-anesthetized littermates (33,899 (21,705–38,354) %ID/g, p = 0.01). [^18^F]FAZA muscle TACs revealed enhanced uptake in KX-anesthetized mice only until 90 min post [^18^F]FAZA injection relative to their IF-anesthetized littermates (**Fig 3D**). Consequently, no significant differences in [^18^F]FAZA-muscle uptake were found when comparing the AUCs in IF- and KX-anesthetized animals (IF-air: 10,973 (10,425–11,461) %ID/g, KX-air: 12,300 (11,374–15,597) %ID/g, p = 0.10).

Tumor [^18^F]FAZA washout kinetics revealed trapping for 1 h after [^18^F]FAZA injection in tumors of KX-anesthetized mice (after the perfusion peak). At 1 h, 94% of the maximum [^18^F]FAZA uptake was reached in tumors of KX-anesthetized mice whereas ~60% of the maximum [^18^F]FAZA uptake was reached in tumors of their IF-anesthetized littermates (**S2A Fig**). In tumors of IF-anesthetized mice, the [^18^F]FAZA activity decreased to 28% 3 h after tracer injection, compared to 56% in the tumors of KX-anesthetized mice. In contrast, we observed comparable [^18^F]FAZA washout in muscle tissue (**S2B Fig**). Tumor volumes in all experimental groups for both static and dynamic measurements were similar (**S3 Fig**).

### 3.3 Tumor and muscle oxygenation and perfusion are impaired by anesthesia

**Fig 4:**
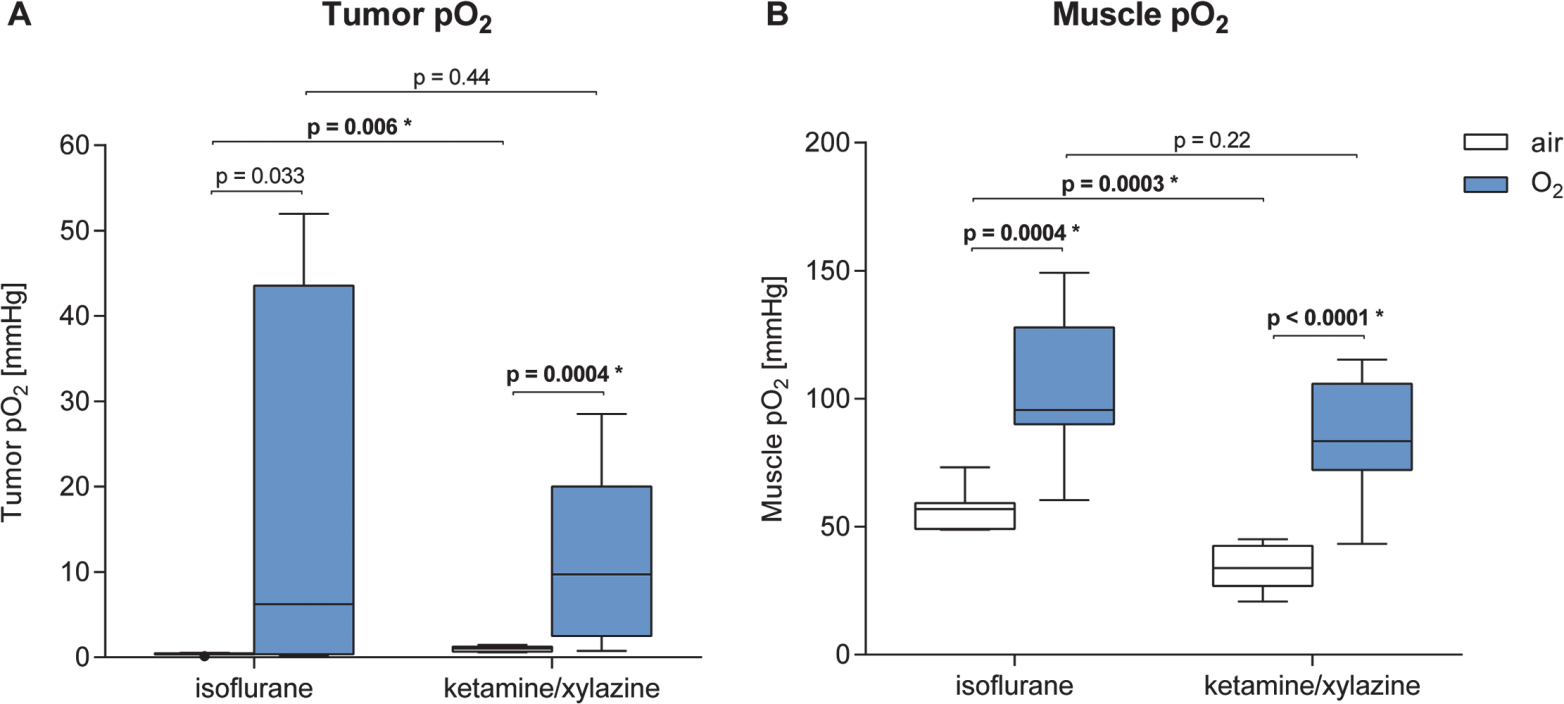
pO_2_-values in tumor (A) and muscle tissue (B). n = 7–9. p-values without asterisks (*) were not significant (after correcting for multiple testing).

To confirm our *in vivo* PET data, we performed invasive tissue oxygen tension (pO_2_) measurements. A tissue oxygen tension- and perfusion-detecting laser-based probe was placed in the center of the CT26 colon carcinomas and into the contralateral *gastrocnemius* muscle (n = 7–9). After placement of the pO_2_ probe, data acquisition was performed for one hour. The obtained values were averaged over the 60-minute measurement period. During the measurement period, we detected constant tissue oxygen tension values in carcinomas and muscles with the exception of the oxygen-breathing mice, in which we measured enhanced pO_2_ values only within the first 20 min. We observed a tendency toward lower tumor tissue oxygen tension values in the carcinomas of IF-anesthetized mice compared to their KX-anesthetized littermates (**Fig 4A and Table 2B**) regardless of whether the mice were breathing air (IF-air: 0.45 mmHg vs. KX-air: 1.01 mmHg, p = 0.006*) or oxygen (IF-O_2_: 6.25 mmHg vs. KX-O_2_: 9.73 mmHg, p = 0.44 n.s.). The tumor tissue oxygen tension values were higher in oxygen-breathing mice compared to air-breathing mice anesthetized with IF (IF-O_2_: 6.25 mmHg vs. IF-air: 0.45 mmHg, p = 0.033 n.s.) or KX (KX-O_2_: 9.73 mmHg vs. KX-air: 1.01 mmHg, p = 0.0004*).

Additionally, we investigated differences in muscle tissue oxygen tension values in mice as a consequence of IF or KX anesthesia as well as of breathing air or oxygen (**Fig 4B**). Muscle tissue oxygen tension values increased in IF-anesthetized mice compared to KX-anesthetized animals regardless of whether the mice were breathing air (IF-air: 56.85 mmHg vs. KX-air: 33.79 mmHg, p = 0.0003*) or oxygen (IF-O_2_: 95.64 mmHg vs. KX-O_2_: 83.48 mmHg, p = 0.22 n.s.). Muscle tissue oxygen tension values were significantly higher in oxygen-breathing mice (IF-O_2_: 95.64 mmHg, KX-O_2_: 83.48 mmHg) compared to their air-breathing littermates (IF-air: 56.85 mmHg, p = 0.0004*, KX-air: 33.79 mmHg, p < 0.0001*) regardless of whether the mice underwent IF or KX anesthesia (**Fig 4B**).

**Fig 5:**
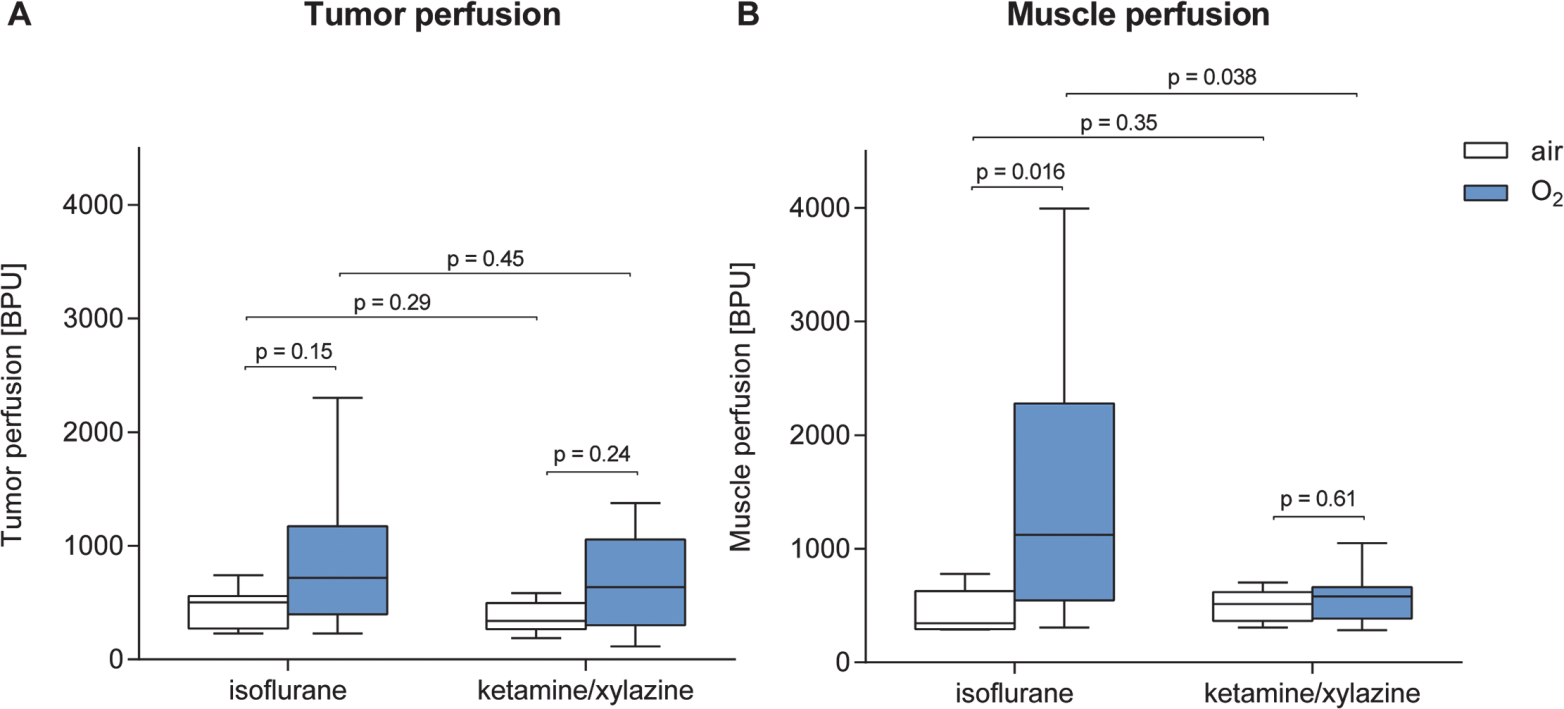
Tumor (A) and muscle (B) perfusion. n = 7–9. p-values without asterisks (*) were not significant (after correcting for multiple testing).

To clarify whether the increased muscle tissue oxygen tension values and decreased [^18^F]FAZA uptake in the tumors of IF-anesthetized mice were the consequence of altered perfusion, we performed *in vivo* laser Doppler measurements, as it was possible to perform simultaneous tissue oxygen tension and perfusion measurements with our probe. We identified a tendency toward increased perfusion of tumors of oxygen-breathing mice compared to tumors of air-breathing mice regardless of whether the mice were anesthetized with IF or KX (IF-air: 503 blood perfusion units (BPU), IF-O_2_: 718 BPU, p = 0.15 n.s., KX-air: 340 BPU, KX-O_2_: 636 BPU, p = 0.24 n.s., **Fig 5A**). These results, although not statistically significant, were consistent with increased pO_2_-values (**Fig 4A**). Focusing on systemic effects, we measured a significant, up to threefold, increase in muscle perfusion in IF-anesthetized oxygen-breathing mice when compared to IF-anesthetized air-breathing mice (IF-O_2_: 1122 BPU vs. IF-air: 345 BPU, **Fig 5B**) and their air- or oxygen-breathing KX-anesthetized littermates (KX-O_2_: 582 BPU, KX-air: 514 BPU). In contrast, muscle perfusion values in KX-anesthetized mice were found to be equivalent regardless of whether the mice were breathing air or oxygen (**Fig 5B**).

### 3.4 Impact of oxygen challenges on tumor and muscle oxygenation

**Fig 6:**
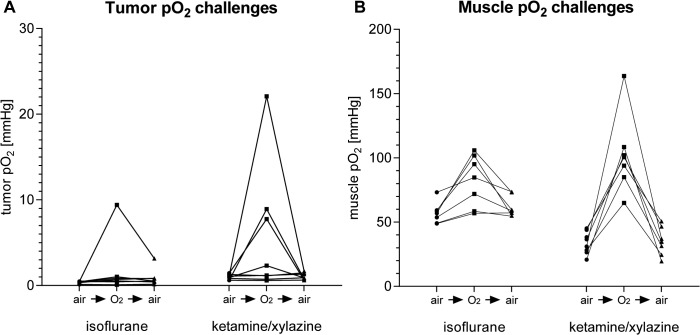
Tumor (A) and muscle (B) pO_2_ values of mice breathing air for 60 min, followed by 10-min oxygen and air challenges. Data from individual mice are connected by a line. n = 7–9.

We further analyzed pO_2_-value responses in tumor and muscle tissue after changing the breathing gas from air to oxygen. Thus, after obtaining a one-hour baseline measurement of spontaneously air-breathing mice under IF or KX anesthesia, we applied a 10-minute oxygen challenge followed by a 10-minute period of air-breathing according to the protocol in **Table 1.** In the carcinomas, the oxygen challenge resulted in a variable increase of intratumoral pO_2_ values in both IF- and KX-anesthetized mice (**Fig 6A**). In line with this finding, the oxygen challenges resulted in significantly increased muscle pO_2_ values regardless of whether the mice underwent IF or KX anesthesia (**Fig 6B,** n = 7–9). Importantly, after switching back to air-breathing, the pO_2_ values in the tumor and muscle tissues decreased within seconds or a few minutes to the baseline values (**Fig 6A and 6B**). Hence, the pO_2_ values in the tumor and muscle tissue responded very quickly to changes in the breathing gas (**S4 Fig**).

### 3.5 Anesthesia and breathing gas impact heart and breathing rates and blood pressure

We investigated the impact of our anesthesia and breathing regimes on the heart and breathing rates and the blood pressure of the mice. In this context, it is important to mention that we had no tools to detect the heart rate and the blood pressure in conscious mice.

**Fig 7:**
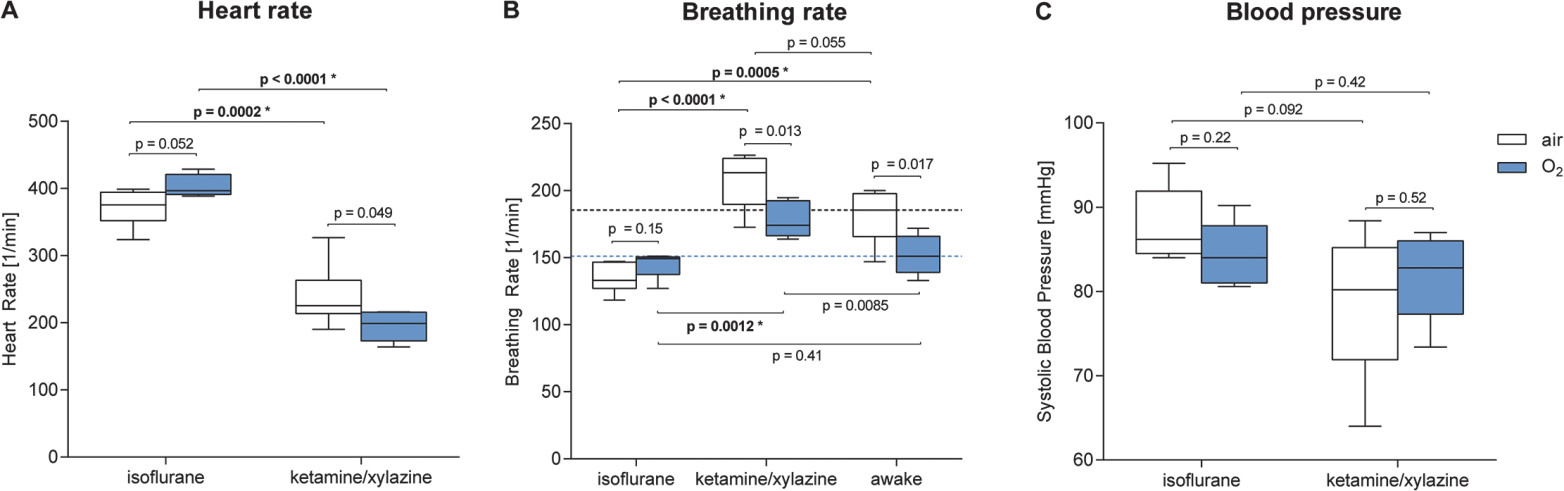
Heart rate (A), breathing rate (B) and systolic blood pressure (C) of conscious mice or their littermates under isoflurane or ketamine/xylazine anesthesia breathing air or oxygen. p-values without asterisks (*) were not significant (after correcting for multiple testing).

We found that the heart rate of IF-anesthetized mice was significantly increased compared to that of their KX-anesthetized littermates irrespective of the breathing gas (IF-air: 376/min vs. KX-air: 225/min, p = 0.0002*, IF-O_2_: 397/min vs. KX-O_2_: 199/min, p < 0.0001*, **Fig 7A and Table 2C**). The heart rate tended to be higher in air-breathing KX-anesthetized mice compared to their oxygen-breathing littermates (KX-air: 225/min vs. KX-O_2_: 199/min, p = 0.049 n.s.), whereas the opposite tendency was observed in mice under IF anesthesia (IF-air: 376/min vs. IF-O_2_: 397/min, p = 0.052 n.s.).

Next, we investigated the breathing rate of individual mice using a whole-body plethysmograph, a breathing pad and a spirometer. This experimental setup enabled us to detect the breathing rate in conscious as well as anesthetized air- or oxygen-breathing mice. In summary, when compared to conscious mice, we found a reduced breathing rate in IF-anesthetized mice and an increased breathing rate in KX-anesthetized mice (**Fig 7B**, n = 5–6). This phenomenon was more pronounced in air-breathing mice. As physiologically expected, breathing oxygen caused a reduction in the breathing rate of both conscious (air: 186/min (166/min–198/min), vs. O_2_: 151/min (139/min–166/min), p = 0.017 n.s.) and KX-anesthetized mice (KX-air: 213/min vs. KX-O_2_: 174/, p = 0.013 n.s.). This was absent in IF-anesthetized mice, in which we observed the opposite tendency (IF-air: 133/min vs. IF-O_2_: 149/min, p = 0.15 n.s.).

Supporting our findings of reduced pO_2_-values in the muscle tissue of KX-anesthetized mice (**Fig 4A and 4B**), the systolic blood pressure tended to be lower in air-breathing KX-anesthetized mice than in their IF-anesthetized littermates (KX-air: 80 mmHg vs. IF-air: 86 mmHg, p = 0.092 n.s., n = 5, **Fig 7C and Table 2C**). Similar blood pressure values were observed in oxygen-breathing KX- and IF-anesthetized animals (KX-O_2_: 83 mmHg vs. IF-O_2_: 84 mmHg, p = 0.42 n.s.).

## Discussion

In this comprehensive study, we addressed the impact of IF and KX anesthesia and air- or oxygen-breathing on *in vivo* tumor hypoxia measurements using non-invasive and invasive techniques. As previous studies have produced controversial results [15, 18, 19], we investigated the impact of the anesthetics and of air- or oxygen-breathing on tumor and muscle perfusion as well as on basic physiological parameters such as heart rate, breathing rate and systolic blood pressure.

Our studies revealed evidence for increased muscle oxygenation when using IF instead of KX as anesthetic regimen (**Fig 4A and 4B**). We observed a higher [^18^F]FAZA uptake and a reduced tracer clearance in the CT26 carcinomas of KX-anesthetized mice compared to their IF-anesthetized littermates, as further supported by dynamic [^18^F]FAZA TAC analysis. Our results conflict with the pO_2_ probe measurements reported by *Baudelet* and *Gallez* [19]. On the contrary, *Kersemans et al*. did not identify any differences in the T/M-ratios between IF and KX-anesthetized mice using [^18^F]FMISO PET imaging, but they did not present *in vivo* %ID/g values [18]. *Erhardt et al*. and *Sumitra et al*. reported lower arterial pO_2_ values in KX-anesthetized mice when compared to IF-anesthetized animals, which could additionally contribute to lower pO_2_ values in the muscle tissue [26, 27]. Interestingly, other investigators reported that KX anesthesia also results in lower oxygenation values in other tissues, e.g., the brain and the skin, when compared to IF anesthesia [28, 29].

The underlying mechanism for reduced systemic oxygenation in KX-anesthetized mice compared to IF-anesthetized mice is not yet completely understood. *Baudelet* and *Gallez* reported decreased tumor perfusion in KX-anesthetized mice [19]. In addition, our studies revealed a tendency toward reduced systolic blood pressure and heart rate in mice that underwent KX anesthesia, supporting these findings (**Fig 7**). We therefore hypothesize that the lower muscle tissue oxygen tension values and the increased tumoral uptake of [^18^F]FAZA in the KX-anesthetized mice compared to the IF-anesthetized animals might be caused by reduced peripheral perfusion combined with reduced blood oxygenation. In contrast, the median tumor tissue oxygen tension values were higher in KX-anesthetized compared to IF-anesthetized animals. This might have been caused by a low-ceiling effect, or, even more importantly, by the fact that our pO_2_ probe focuses on extracellular oxygenation, whereas [^18^F]FAZA focuses on intracellular oxygenation.

Our investigations of the impact of oxygen- or air-breathing on tumor and muscle oxygenation revealed increased tumor and muscle tissue oxygen tension values in oxygen-breathing mice under both anesthesia regimes (**Fig 4**). In contrast, our PET studies did not show reduced %ID/g or increased tumor tracer clearance in oxygen-breathing mice compared to air-breathing mice, irrespective of the anesthetic regimen (**Fig 2A**). However, oxygen-breathing in KX-anesthetized mice resulted in decreased T/M-ratios at 3 h p.i. compared to their air-breathing littermates, which was mainly caused by reduced [^18^F]FAZA muscle clearance and not by enhanced tumor tracer uptake. Previous studies have shown that oxygen breathing in ketamine/xylazine-anesthetized mice might cause respiratory acidosis possibly affecting the entire organism and should therefore be avoided [20].

The combination of the findings from our own and previous investigations strengthens the hypothesis that oxygen-breathing instead of air-breathing reduces T/M-ratios in animals that were anesthetized exclusively for the PET acquisition time [15, 18, 30], while this effect was absent when animals were anesthetized throughout the entire tracer uptake and PET acquisition period (**S3 Table,** [18]). This leads to the assumption that oxygen-breathing can severely reduce hypoxia tracer uptake mainly when the mice are awake during the hypoxia tracer uptake phase. Because %ID/g-values were not consistently reported by all investigators, we cannot ascertain whether these effects were mainly caused by altered hypoxia tracer uptake within the tumor and/or by altered clearance from the muscle tissue. As this finding was not explicitly included in our investigational design, further studies are necessary to clarify our hypothesis.

As we cannot explain the difference between the %ID/g values and the tissue oxygen tension measurements in the tumor tissue, we hypothesize that these discrepancies might be caused by the fact that [^18^F]FAZA reflects intracellular hypoxia [31], while our pO_2_ measurements are more likely related to the extracellular tissue concentration of oxygen. In addition, the invasive nature of oxygen-probe measurements might change the tumor microenvironment [32]. We used the CT26 colon carcinoma model because these tumors are largely homogeneous and non-necrotic (**S5 Fig**). The homogeneous nature of our CT26 colon carcinomas is also apparent in our PET images, which indicate homogeneous [^18^F]FAZA uptake (**Fig 1**) It remains a very difficult task to clarify the origin of the reported discrepancies in 2-nitroimidazole imaging studies throughout the literature, as a calibrated, absolute and reliable correlative hypoxia measurement technique is still not available after 20 years of hypoxia imaging.

Interestingly, anesthetic agent effects on PET imaging results have also been reported for other PET tracers. In addition, *Matsumura* et al. reported that different anesthetics and tracer injection regimes (anesthesia prior to vs. post tracer injection) impair the uptake of [^18^F]-fluoro-2-deoxy-D-glucose ([^18^F]FDG) in rat brain [33]. Interestingly, total brain uptake in rats that were anesthetized with ketamine was not significantly different from that in conscious rats, whereas KX-anesthesia yielded a significant decrease in [^18^F]FDG uptake in cortex compared to that in conscious rats [33]. These results, when taken together with our results and those of recent studies [30], indicate that the choice of anesthetic agent and the timing of the anesthesia induction might cause more dramatic effects on our PET data than some other interventions, e.g., therapeutic studies.

We conclude that the anesthesia and breathing gas regimens can severely impair non-invasive PET and invasive pO_2_ probe tumor hypoxia measurements. It is uncertain whether these effects are directly caused by the anesthetics, or if the observed differences arise because an effect of the anesthesia on cardiovascular regulation has an impact on tissue perfusion. Even this comprehensive investigation failed to elucidate clear mechanisms. Thus, anesthetics affect the entire organism; it should be noted that the metabolic and pharmacodynamic properties of the anesthetics used might impair the metabolic and pharmacodynamic properties of the PET tracer. Additional, 3 h dynamic scans were performed with air-breathing mice that were anesthetized with IF or KX, accordingly (n = 4). Both groups were injected with a comparable activity of [^18^F]FAZA (IF-air: 12.37 ± 0.12 MBq, KX-air: 12.21 ± 0.12 MBq). However, strengthening the hypothesis regarding the impact of anesthesia, we recently showed that anesthesia-induced effects are also evident in 3'-[^18^F]fluoro-3'-deoxythymidine ([^18^F]FLT)-tumor-PET studies [14]. A recent report showed that the choice of the anesthetic agent as well as the mode of anesthesia (regional or general) might even impact the clinical outcome in patients undergoing surgical carcinoma resection [34].

Tumor hypoxia measurements have been proven valuable in the clinical setting [3]. However, to date, preclinical tumor hypoxia measurements seem to be flawed by too many confounders to be comparable. These potentially interfering factors are not limited to the tumor cell line and include different mouse species, ambient baseline conditions (temperature, animal housing, etc.), anesthetic agent (including dosing) and breathing gas. In summary, we are convinced that oxygen-breathing during hypoxia investigations does not reflect the “physiologic” hypoxia state and should in general be replaced by air-breathing. Because anesthesia is required for most PET investigations, it should be limited to the PET acquisition time, as this limits the potential impact on hypoxia measurements. However, as most commonly used anesthetics in the preclinical setting are not feasible for patients, anesthetics such as propofol or midazolam should be evaluated in hypoxia PET studies to enable the transition of preclinical studies into the clinic (e.g. children and mentally disabled). Magnetic resonance-based tools might help to overcome some PET-specific limitations because they do not rely on tracer whole body pharmacodynamics and pharmacokinetics that might be impaired by anesthetics. However, we cannot exclude anesthesia-related influences on MRI measurements that arise from metabolic changes or other anesthetic side effects. Generally speaking, the study protocol for hypoxia measurements in basic research applications should be unified to facilitate comparative studies among different tumor entities.

## Conclusion

This comprehensive study uncovered that the anesthesia regimes commonly used in the preclinical setting exert a strong impact on a variety of parameters, such as [^18^F]FAZA uptake and tissue oxygen tension (pO_2_) within tumor and muscle tissue.

KX reveals superior signal-to-noise ratios in PET hypoxia imaging when compared to IF, the most widely used preclinical anesthetic. The enhanced T/M-ratios found in KX-anesthetized mice compared to their IF-anesthetized littermates are associated with significantly increased [^18^F]FAZA uptake (%ID/g) in the CT26 colon carcinomas. Surprisingly, the [^18^F]FAZA uptake in tumors of KX-anesthetized and air-breathing mice yielded a much higher variance than IF-anesthetized mice.

We recommend air breathing because air breathing reduces tissue oxygen tension and enhances T/M-ratios in KX-anesthetized CT26 colon carcinoma-bearing mice. Moreover, invasive tissue oxygen tension and perfusion measurements yield a low variance in tumors of air-breathing mice and a high variance in tumors of oxygen-breathing mice, irrespective of the anesthesia regime.

In addition, we elucidated anesthesia-related impairments of mouse physiology: IF anesthesia yielded increased heart rate and decreased breathing rate compared to KX anesthesia, and no significant differences in blood pressure were observed.

We conclude that the differential effects of these anesthetics on mouse physiology and metabolism might severely impair oxygenation status and, consequently, tumor hypoxia and should therefore not be underestimated. The standardization of anesthetics in preclinical use as well as the preclinical establishment of agents in clinical use might facilitate animal research and allow for a more rapid clinical translation of preclinical results.

## Supporting Information

S1 Fig Whole body plethysmography. (EPS)

S2 Fig Dynamic washout. (EPS)

S3 Fig Comparison of tumor volumes. (EPS)

S4 Fig Tumor and muscle pO_2_ dynamics (EPS)

S5 Fig CT26 histology. (EPS)

S1 Table Literature review of the impact of different anesthetics on physiologic parameters. Arrows indicate changes relative to awake animals. BP = blood pressure, CI = cardiac index, EF = ejection fraction, MAP = mean arterial pressure. Extended by the authors [14].
(PDF)

S2 Table %ID/cc values in the tumor and in the muscle. Provided at 1h, 2h and 3h after [^18^F]FAZA injection with median, 25% percentile (25%P), 75% percentile (75%P) and group size (n).
(PDF)

S3 Table Literature review of the impact of air or oxygen breathing on PET tracer uptake. (PDF)
