# Supplementary material for: A Comparative pO2 Probe and [18F]-Fluoro-Azomycinarabino-Furanoside ([18F]FAZA) PET Study Reveals Anesthesia-Induced Impairment of Oxygenation and Perfusion in Tumor and Muscle
Source: PLoS One. 2015 Apr 22;10(4):e0124665. doi: 10.1371/journal.pone.0124665 (PMC4406741; doi:10.1371/journal.pone.0124665)
Supplement: S1 Table — Arrows indicate changes relative to awake animals. BP = blood pressure, CI = cardiac index, EF = ejection fraction, MAP = mean arterial pressure. Extended by the authors [14]. (PDF) [file pone.0124665.s006.pdf]

S1 Table. Literature review of the impact of different anesthetics on physiologic parameters.

Arrows indicate changes relative to awake animals. BP = blood pressure, CI = *cardiac index*, EF = ejection fraction, MAP = mean arterial pressure. Extended by the authors [1].

	ketamine/xylazine		isoflurane	awake
	ketamine	xylazine		
**mode of action**	NMDA receptor antagonist [2]	selective α_2 adrenoceptor agonist [3]	$GABA_A$ agonist [4]	
**anesthesia**	relaxation [5]<br>hypnosis [5]<br>analgesia [5-7]		sedation [4]<br>relaxation [4]	
**respiratory rate [1/min]**	↓ [5]		↓ [5,8]<br>(dose dependent)	195.4 ± 3.3 [5]<br>288 [9]
**blood pressure [mmHg]**	↔ / ↑ [7]		MAP: 79 ± 3 [10,11]<br>↓ [12]	BP: 106 ± 2 [13]<br>BP: 96.1 [14]<br>BP: 126 [15]<br>MAP: 105 ± 3 [11]<br>MAP: 92 ± 5 [16]
	In combination: ↓ [5]<br>BP: 46 ± 5 mmHg [11]			
**peripheral resistance**	↔ [17,18]		arteriole: ↔ [19]<br>peripheral resistance: ↓	
**heart: inotropy**	↓ [20], EF ↓ [21]<br>CI: -63 % [11]		CI: -24 % to -42 % [11]	CI: 0.46 ± 0.02 mL·min^{-1}·g^{-1}
**heart: chronotropy [1 / min]**	↓ [22]	↓ [23]	516–527 [8]<br>(dose dependent)<br>↔ 486 ± 13 [10]	508.6 ± 19.2 [5]<br>503 ± 52 [13]<br>658 ± 9 [21]<br>573 ± 14 [16]<br>*537* [24]<br>468 [14]<br>569 [15]
	↓ [20]<br>293 ± 19 [21]<br>308 ± 14 [25]			
**arterial blood gases [mmHg]** pO_2	97.3 ± 4.7 = ↓ [5] ($p > 0.05$)		130 ± 19 [10]	111.7 ± 5.2 [5]
pCO_2	41.0 ± 1.4 = ↑ [5]		35.6 ± 4.7 [10]	26.5 ± 3.5 [5]
pH	7.1 ± 0.02 = ↓ [5]		7.29 ± 0.06 [10]	7.285 ± 0.03 [5]
**miscellaneous**		diuresis [26-28]<br>hyperglykaemia [15,29,30]	Reduced glomerular filtration rate [31,32]	

Literature

1. Fuchs K, Kukuk D, Mahling M, Quintanilla-Martinez L, Reischl G, et al. (2013) Impact of anesthetics on 3'-[18F]fluoro-3'-deoxythymidine ([18F]FLT) uptake in animal models of cancer and inflammation. Mol Imaging 12: 277-287.

2. Hirota K, Lambert DG (1996) Ketamine: its mechanism(s) of action and unusual clinical uses. Br J Anaesth 77: 441-444.

3. Flecknell P (2009) Laboratory Animal Anaesthesia: Elsevier/Academic Press.

4. Campagna JA, Miller KW, Forman SA (2003) Mechanisms of actions of inhaled anesthetics. N Engl J Med 348: 2110-2124.

5. Erhardt W, Hebestedt A, Aschenbrenner G, Pichotka B, Blümel G (1984) A comparative study with various anesthetics in mice (pentobarbitone, ketamine-xylazine, carfentanyl-etomidate). Research in Experimental Medicine 184: 159-169.

6. White GL, Holmes DD (1976) A comparison of ketamine and the combination ketamine-xylazine for effective surgical anesthesia in the rabbit. Laboratory animal science 26: 804-806.

7. Clarke KW, Trim CM, Hall LW (2014) General pharmacology of the injectable agents used in anaesthesia. Veterinary Anaesthesia. Edinburgh: Elsevier Ltd. pp. 135-153.

8. Kober F, Iltis I, Cozzone PJ, Bernard M (2005) Myocardial blood flow mapping in mice using high-resolution spin labeling magnetic resonance imaging: influence of ketamine/xylazine and isoflurane anesthesia. Magn Reson Med 53: 601-606.

9. Berndt A, Leme AS, Williams LK, Von Smith R, Savage HS, et al. (2011) Comparison of unrestrained plethysmography and forced oscillation for identifying genetic variability of airway responsiveness in inbred mice. Physiol Genomics 43: 1-11.

10. Szczesny G, Veihelmann A, Massberg S, Nolte D, Messmer K (2004) Long-term anaesthesia using inhalatory isoflurane in different strains of mice-the haemodynamic effects. Lab Anim 38: 64-69.

11. Janssen BJ, De Celle T, Debets JJ, Brouns AE, Callahan MF, et al. (2004) Effects of anesthetics on systemic hemodynamics in mice. Am J Physiol Heart Circ Physiol 287: H1618-1624.

12. Zuurbier CJ, Emons VM, Ince C (2002) Hemodynamics of anesthetized ventilated mouse models: aspects of anesthetics, fluid support, and strain. American journal of physiology Heart and circulatory physiology 282: H2099-2105.

13. Desai KH, Sato R, Schauble E, Barsh GS, Kobilka BK, et al. (1997) Cardiovascular indexes in the mouse at rest and with exercise: new tools to study models of cardiac disease. Am J Physiol 272: H1053-1061.

14. Berthonneche C, Peter B, Schupfer F, Hayoz P, Kutalik Z, et al. (2009) Cardiovascular response to beta-adrenergic blockade or activation in 23 inbred mouse strains. PLoS One 4: e6610.

15. The Jackson Laboratory (2012) Multi-system analysis of physiology on 7 inbred strains of mice. MPD:Jaxwest1. Mouse Phenome Database web site, The Jackson Laboratory, Bar Harbor, Maine USA. http://phenome.jax.org.

16. Butz GM, Davisson RL (2001) Long-term telemetric measurement of cardiovascular parameters in awake mice: a physiological genomics tool. Physiol Genomics 5: 89-97.

17. Schwartz DA, Horwitz LD (1975) Effects of ketamine on left ventricular performance. J Pharmacol Exp Ther 194: 410-414.

18. Gooding JM, Dimick AR, Tavakoli M, Corssen G (1977) A physiologic analysis of cardiopulmonary responses to ketamine anesthesia in noncardiac patients. Anesth Analg 56: 813-816.

19. Meyer JU, Intaglietta M (1986) Measurement of the dynamics of arteriolar diameter. Ann Biomed Eng 14: 109-117.

20. Roth DM, Swaney JS, Dalton ND, Gilpin EA, Ross J, Jr. (2002) Impact of anesthesia on cardiac function during echocardiography in mice. Am J Physiol Heart Circ Physiol 282: H2134-2140.

21. Yang XP, Liu YH, Rhaleb NE, Kurihara N, Kim HE, et al. (1999) Echocardiographic assessment of cardiac function in conscious and anesthetized mice. Am J Physiol 277: H1967-1974.

22. Mitchell GF, Jeron A, Koren G (1998) Measurement of heart rate and Q-T interval in the conscious mouse. Am J Physiol 274: H747-751.

23. Clarke KW, Trim CM, Hall LW (2014) Prinicples of sedation, anticholinergic agents, and principles of premedication. Veterinary Anaesthesia. Edinburgh: Elsevier Ltd. pp. 79-100.

24. Hampton T, Paigen B, Seburn K (2001) Electrocardiography in 17 inbred strains of mice. MPD:Hampton1. Mouse Phenome Database web site, The Jackson Laboratory, Bar Harbor, Maine USA. http://phenome.jax.org.

25. Chaves AA, Weinstein DM, Bauer JA (2001) Non-invasive echocardiographic studies in mice: influence of anesthetic regimen. Life Sciences 69: 213-222.

26. Cabral AM, Varner KJ, Kapusta DR (1997) Renal excretory responses produced by central administration of opioid agonists in ketamine and xylazine-anesthetized rats. J Pharmacol Exp Ther 282: 609-616.

27. Cabral AD, Kapusta DR, Kenigs VA, Varner KJ (1998) Central alpha2-receptor mechanisms contribute to enhanced renal responses during ketamine-xylazine anesthesia. The American journal of physiology 275: R1867-1874.

28. Miller JH, McCoy KD, Coleman AS (2001) Renal actions of the alpha2-adrenoceptor agonist, xylazine, in the anaesthetised rat. N Z Vet J 49: 173-180.

29. Abdel el Motal SM, Sharp GW (1985) Inhibition of glucose-induced insulin release by xylazine. Endocrinology 116: 2337-2340.

30. Pomplun D, Mohlig M, Spranger J, Pfeiffer AF, Ristow M (2004) Elevation of blood glucose following anaesthetic treatment in C57BL/6 mice. Horm Metab Res 36: 67-69.

31. Chou YP, Huang WC, Chang CL, Lin CY (1990) [Renal effect of isoflurane]. Ma Zui Xue Za Zhi 28: 410-418.

32. Mercatello A (1990) [Changes in renal function induced by anesthesia]. Ann Fr Anesth Reanim 9: 507-524.
