# Supplementary material for: A Comparative pO2 Probe and [18F]-Fluoro-Azomycinarabino-Furanoside ([18F]FAZA) PET Study Reveals Anesthesia-Induced Impairment of Oxygenation and Perfusion in Tumor and Muscle
Source: PLoS One. 2015 Apr 22;10(4):e0124665. doi: 10.1371/journal.pone.0124665 (PMC4406741; doi:10.1371/journal.pone.0124665)
Supplement: S2 Table — Provided at 1h, 2h and 3h after [18F]FAZA injection with median, 25% percentile (25%P), 75% percentile (75%P) and group size (n). (PDF) [file pone.0124665.s007.pdf]

S2 Table. %ID/cc values in the tumor and in the muscle.

Provided at 1h, 2h and 3h after [^{18}F]FAZA injection with median, 25% percentile (25%P), 75% percentile (75%P) and group size (n).

	Air				Oxygen			
	**Median**	**25%P**	**75%P**	**n**	**Median**	**25%P**	**75%P**	**n**
**1h: Tumor**								
Isoflurane	1.73	1.41	1.83	7	1.66	1.37	2.00	10
Ketamine/xylazine	3.95	2.38	4.47	12	3.33	3.01	3.81	8
**1h: Muscle**								
Isoflurane	1.30	1.17	1.56	7	1.40	1.28	1.60	10
Ketamine/xylazine	1.72	1.40	2.05	12	2.05	1.74	2.14	8
**2h: Tumor**								
Isoflurane	1.03	0.85	1.26	7	1.09	0.97	1.88	7
Ketamine/xylazine	3.51	1.81	4.19	12	3.45	2.82	3.92	7
**2h: Muscle**								
Isoflurane	0.69	0.68	0.80	7	0.88	0.71	0.89	7
Ketamine/xylazine	0.87	0.60	1.11	12	1.18	0.99	1.44	7
**3h: Tumor**								
Isoflurane	0.67	0.48	1.03	8	0.98	0.60	1.26	9
Ketamine/xylazine	3.13	1.49	4.30	12	3.35	2.57	3.62	8
**3h: Muscle**								
Isoflurane	0.41	0.33	0.46	8	0.51	0.48	0.76	9
Ketamine/xylazine	0.49	0.35	0.60	12	0.76	0.62	0.94	8
