# Supplementary material for: A Comparative pO2 Probe and [18F]-Fluoro-Azomycinarabino-Furanoside ([18F]FAZA) PET Study Reveals Anesthesia-Induced Impairment of Oxygenation and Perfusion in Tumor and Muscle
Source: PLoS One. 2015 Apr 22;10(4):e0124665. doi: 10.1371/journal.pone.0124665 (PMC4406741; doi:10.1371/journal.pone.0124665)
Supplement: S3 Table — (PDF) [file pone.0124665.s008.pdf]

S3 Table. Literature review of the impact of air or oxygen breathing on PET tracer uptake.

	consciousness	Tumor	Mouse model	Tracer	Tumor/muscle-ratio
Anesthetized throughout uptake and data acquisition					
Our data	Isoflurane (1.5%)	CT26	Female BALB/c	[^{18}F]FAZA	O_2 = Air (3h) ~
Our data	Ketamine/Xylazine	CT26	Female BALB/c	[^{18}F]FAZA	O_2 = Air (3h) ~
Kersemans et al. 2011	Isoflurane (1.5%)	CaNT s.c.	Female CVA	[^{18}F]FMISO	O_2 = Air (2h) ~
Anesthetized only for data acquisition					
Piert et al. 2005	Ketamine/Xylazine for data acquisition only	A431 s.c.	Female Swiss nude	[^{18}F]FAZA	O_2 < Air (2h, tumor/background)
Kersemans et al. 2011	Not anesthetized (Dissection)	CaNT s.c.	Female CVA	[^{18}F]FMISO	O_2 < Air (2h) *
Maier et al. 2011	Isoflurane (1.5%) anesthetized for data acquisition only	CT26	Female BALB/c	[^{18}F]FAZA	O_2 < Air (3h) *

* significant
~ Tested for difference, but no significant difference detected
[1] not tested by the authors. t-Test $p < 0.0001$ for air (103 ± 13 mmHg, n = 5) vs. Oxygen (434 ± 68 mmHg, n = 5)
